# Correlation between MEK signature and Ras gene alteration in advanced gastric cancer

**DOI:** 10.18632/oncotarget.18182

**Published:** 2017-05-23

**Authors:** Soomin Ahn, Roz Brant, Alan Sharpe, Jonathan R. Dry, Darren R. Hodgson, Elaine Kilgour, Kyung Kim, Seung Tae Kim, Se Hoon Park, Won Ki Kang, Kyoung-Mee Kim, Jeeyun Lee

**Affiliations:** ^1^ Department of Pathology and Translational Genomics, Samsung Medical Center, Sungkyunkwan University School of Medicine, Seoul, Korea; ^2^ AstraZeneca, Oncology Innovative Medicines, Alderley Park, Macclesfield, UK; ^3^ AstraZeneca, Cambridge, UK; ^4^ AstraZeneca, Boston, Massachusetts, USA; ^5^ Department of Medicine, Division of Hematology-Oncology, Samsung Medical Center, Sungkyunkwan University School of Medicine, Seoul, Korea

**Keywords:** gastric cancer, KRAS, MEK, selumetinib

## Abstract

MEK inhibitor (selumetinib) is a potent, orally active inhibitor of MAPK/ERK pathway. It is important to develop an accurate and robust method indicative of RAS pathway activity to stratify potential patients who can benefit from selumetinib treatment in gastric cancer (GC). First, we surveyed the sensitivity to selumetinib in a panel of 22 GC cell lines and correlated with MEK signature to selumetinib sensitivity. Next, we analyzed MEK signature via nanostring assay in two Asian cohorts using clinical samples (*n* = 218) and then performed a correlative analysis with MEK signature status and *KRAS* genotype in GC. MEK signature was predictive of response of selumetinib in GC cell lines regardless of *KRAS* mutation status. The proportion of high MEK signature by nanostring assay was 6.9% and the proportion of high MEK signature was significantly higher in *KRAS* altered group in a Korean cohort. None of *PIK3CA* altered cases belonged to high MEK signature group. MEK high signature was more prevalent in intestinal type by Lauren classification. The correlation between MEK signature, *KRAS* alteration and treatment response to selumetinib should be validated in prospective clinical trials.

## INTRODUCTION

Selumetinib (AZD6244, ARRY-142886) is a potent, orally active inhibitor of mitogen-activated protein/extracellular signal-regulated kinase (ERK) kinase (MEK)-1/2 that suppresses the pleiotropic output of the RAF/MEK/ERK pathway [1, 2]. The kinase pathway of RAF/MEK/ERK is activated in most human tumors, often through gain-of-function mutations of *RAS* and *RAF* family members [3, 4]. *In vitro* studies demonstrated a tendency toward sensitivity to MEK inhibitors in tumor cell lines harboring *KRAS* or *BRAF* mutations [1–3, 5, 6]. Based on this preclinical evidence, several clinical trials have tested or are testing the efficacy of MEK inhibitors in *KRAS*-mutant lung cancer patients and colorectal cancer [5, 7, 8].

On the other hand, there was no absolute correlation with mutational or phospho-protein markers of BRAF/MEK, RAS, or phosphoinositide 3-kinase (PI3K) activity to MEK inhibitor response in large tumor cell panels of diverse cancer types [2]. An 18-gene signature enabling measurement of MEK functional output independent of tumor genotype has been reported [2]. There have been several studies to show that RAS pathway is activated in the absence of *KRAS* mutation and the RAS pathway signature is superior to *KRAS* mutation status for the prediction of response to RAS pathway inhibitor [9].

The aim of this study was to investigate the clinicopathologic and genomic status, especially *KRAS* status, of gastric cancer (GC) patients according to MEK signature in two Asian cohorts using clinical samples. In this study, we first surveyed the sensitivity to MEK inhibitor in a panel of GC cell lines and correlated with, *RAS* alteration, MEK signature to MEK inhibitor sensitivity. Next, we analyzed MEK signature via nanostring assay in FFPET (Formalin fixed paraffin embedded tissue) samples from advanced GC patients and then performed a correlative analysis with MEK signature status and *KRAS* genotype in GC.

## RESULTS

### MEK signature in GC cell lines

**Figure 1:**
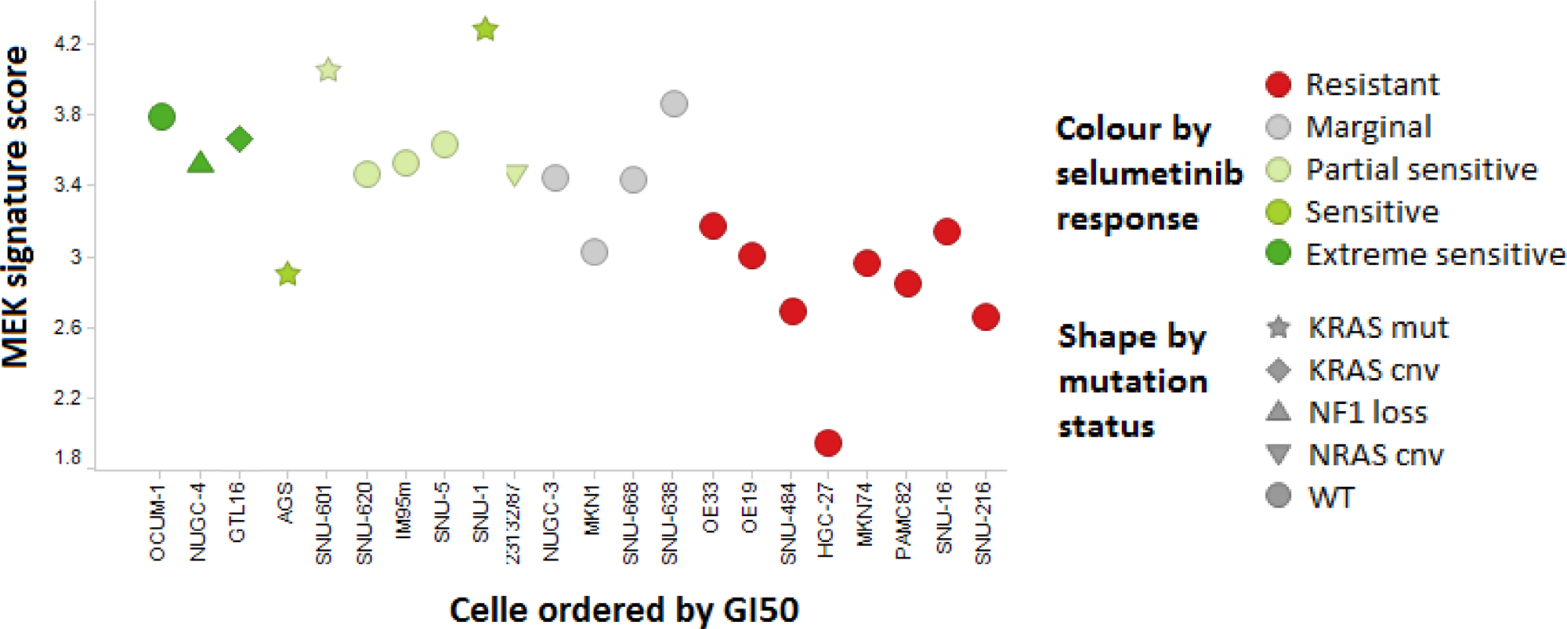
MEK signature and sensitivity to selumetinib in a panel of GC cell lines Cell lines sensitive to MEK inhibition (GI50 under 3 μM; partial if TGI not reached, extreme if TGI < 5 μM) consistently show higher MEK signature score that resistant (GI50 > 20 μM) cell lines, and include all cells with known MEK pathway activating genetic alterations. Few KRAS wild type cell lines (OCUM-1, SNU-620, IM95m, open circles) with high MEK signature were sensitive to selumetinib. SNU-668 cell line was categorized as KRAS wild-type in this figure (KRAS codon 61 mutation).

High MEK signature score is reported [2] to enrich for sensitivity to MEK inhibition in cancer cell lines, low MEK signature score is predictive of resistance, and high “compensatory resistance (Cres)” signature score predictive of resistance in the presence of high MEK signature. In an independent set of 22 cell lines of gastric tumour origin with both RNAseq expression and selumetinib pharmacology, the MEK signature was found similarly predictive of response to selumetinib (ANOVA *p* < 0.00054) (Figure 1). Furthermore, the Cres signature was seen to be predictive of resistance (ANOVA *p* < 0.0068), and the combination (MEK score – Cres score) further separated sensitivity form resistance (ANOVA *p* < 0.00064) (Figure 1). Interestingly, OCUM-1 and SNU-620 cells which are KRAS wild-type but high MEK signature were sensitive to selumetinib.

### MEK signature results according to *KRAS* status in GC specimens

**Figure 2:**
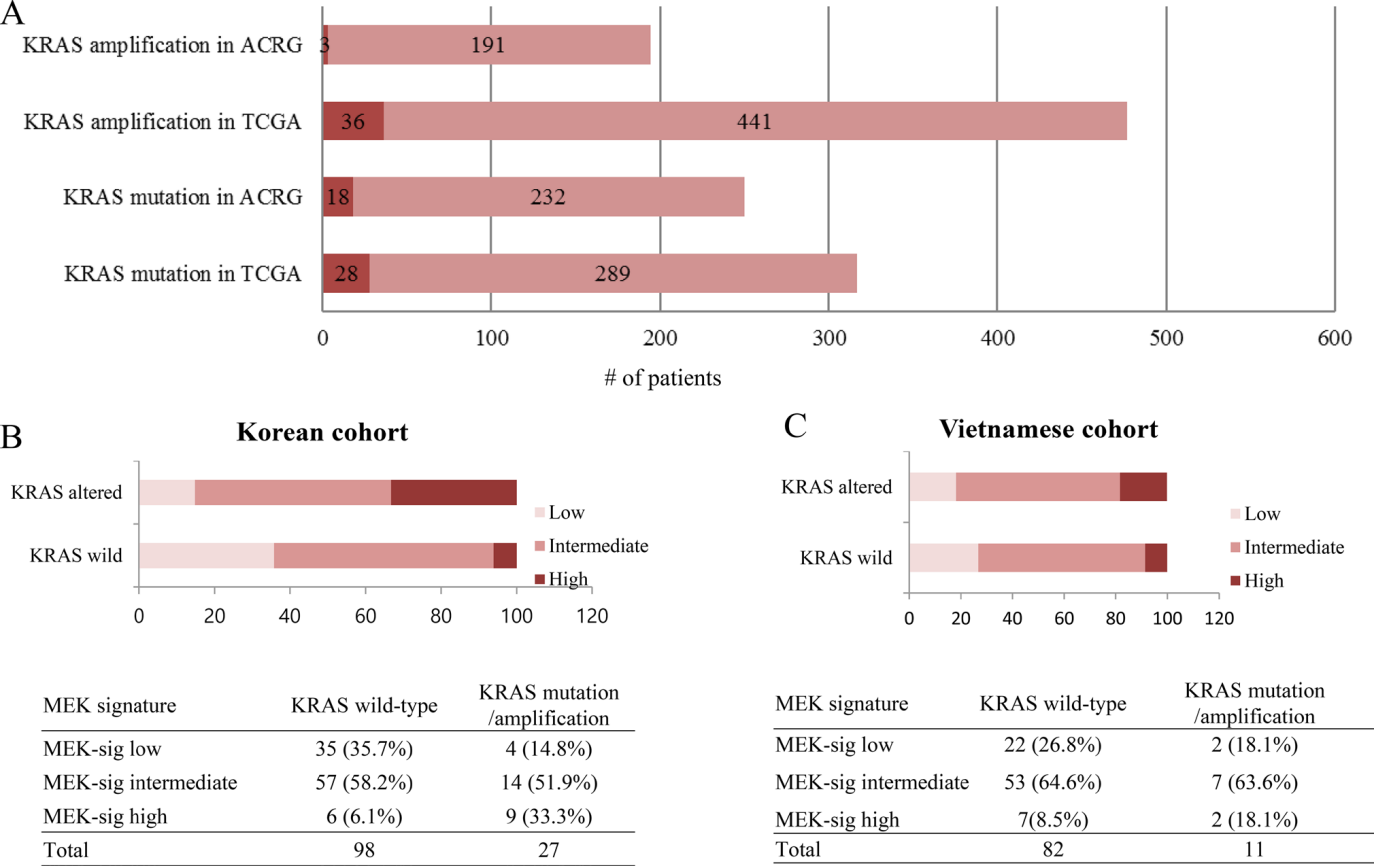
RAS mutation/amplification and distribution of MEK signature in GC (**A**) The incidence of *KRAS* mutation/amplification in ACRG and TCGA. (**B**) Distribution of MEK signature in GC (*N* = 125, Korean). (**C**) Distribution of MEK signature in GC (*N* = 93, Vietnamese).

First, we surveyed the incidence of *KRAS* amplification and mutation status in two large cohorts from previous study [10, 11]. The incidence of *KRAS* amplification was 1.5% (3/191) in the ACRG cohort (all Korean) and 7.5% (36/477) in the TCGA cohort (Figure 2A). The incidence of *KRAS* mutation was 7.2% (18/250) in the ACRG cohort and 8.8% (28/317) in the TCGA cohort.

In total in the Korean cohort, 27 out of 125 patients (21.6%) showed *KRAS* alteration (17 (13.6%) with *KRAS* mutation and 10 (8.0%) with *KRAS* amplification) in a Korean cohort. Detected *KRAS* mutations were as follows; G12C (*n* = 2), G12D (*n* = 9), G12V (*n* = 1), A146P (*n* = 1), A146T (*n* = 1), F156L (*n* = 1), Q61H (*n* = 1), and Q61R (*n* = 1). Of the 125 patients, 15 (12%), 71 (56.9%), and 39 (31.2%) patients were classified as high, intermediate, and low respectively for the MEK gene expression signature score by nanostring. Of 125 patients, 87 patients were prospectively enrolled onto the VIKTORY screening program. In this patient cohort, 6 (6.9%), 50 (57.5%), and 31 (35.6%) patients had high, intermediate, and low MEK signature, respectively.

**Figure 3:**
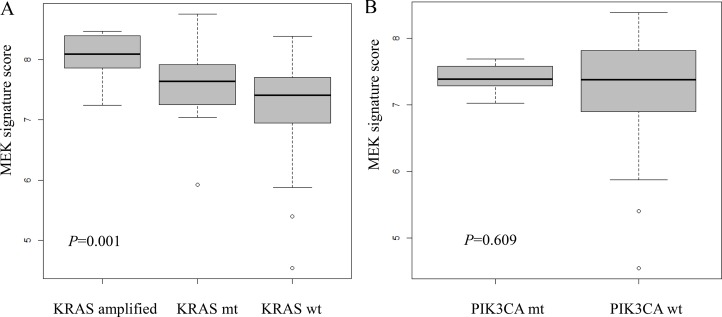
Distribution of MEK signature in Korean cohort according to (**A**) *KRAS* mutation or amplification; (**B**) *PIK3CA* mutation status.

There was a statistically significant difference of distribution in MEK signature group according to *KRAS* status (*P* = 0.001) in the Korean cohort (Figure 2B). For *KRAS* wild-type group (*n* = 98), 35 (35.7%), 57 (58.2%), and 6 (6.1%) cases were classified into low, intermediate, and high MEK signature, respectively. For *KRAS* altered group (*n* = 27), 4 (14.8%), 14 (51.9%), and 9 (33.3%) cases were classified into low, intermediate, and high MEK signature, respectively. The proportion of high MEK signature was significantly higher in the *KRAS* altered group (33.3% in *KRAS* altered group vs 6.1% in *KRAS* wild-type group) (Figure 2B). The mean MEK signature score of *KRAS* wild, mutant, and amplified group was 7.27 (95% confidence interval 7.14–7.41), 7.62 (7.28–7.97), and 8.05 (7.77–8.33) (*P* = 0.01) (Figure 3A).

In the Vietnamese cohort, the proportion of high MEK signature was higher in the *KRAS* altered group (18.1%) compared to *KRAS* wild group (8.5%) although there was no statistical significance (*P* = 0.48) (Figure 2C).

### The association of MEK signature with *BRAF* and *PIK3CA* status

The status of *BRAF* and *PIK3CA* in each MEK signature group is summarized in Table 1. In the VIKTORY screening cohort, there were 7 (8.0%) *PIK3CA* mutant and 1 (1.15%) *PIK3CA* amplified cases. None of 8 *PIK3CA* altered cases belonged to high MEK signature group. The mean MEK signature score of *PIK3CA* wild and altered group was 7.27 (95% confidence interval 7.11–7.43), 7.40 (7.22–7.58) (Figure 3B). There was no statistical significance (*P* = 0.609). *BRAF* mutation was not identified in any of the cases.

**Table 1:** BRAF, and PIK3CA status 87 Korean GC patients

	MEK signature			
	Low (*n* = 31)	Intermediate (*n* = 50)	High (*n* = 6)	Total (*n* = 87)
*BRAF* mutation				
Wild	31 (100%)	50 (100%)	6 (100%)	87
Mutant	0 (0%)	0 (0%)	0 (0%)	0
*PIK3CA* mutation/amplification				
Wild	30 (96.8%)	43 (86.0%)	6 (100%)	79
Mutant	1 (3.2%)	6 (12%)	0 (0%)	7
Amplified	0 (0%)	1 (2%)	0 (0%)	1

On the other hand, *PIK3CA* mutation was detected in 13 (14.0%) out of 93 patients in a Vietnamese cohort. Likewise, none of these patients belonged to the MEK high group. In terms of *BRAF* mutation, 7 (7.5%) *BRAF* mutated patients were identified and 1 out of 7 *BRAF* mutated patients belonged to the MEK high group. There was no association between MEK signature score and BRAF status (*data not shown*).

### Clinicopathologic features of MEK low, intermediate, and high signature group

Table 2 summarizes clinicopathologic characteristics for the Korean cohort according to the MEK signature status. Of note, MEK high signature was more prevalent in well-differentiated and moderately-differentiated tumor type (Table 2). There was a higher percentage of differentiated tumors in the high MEK group (46.7%) compared to the low (12.9%) and intermediate (29.6%) MEK groups (*P* = 0.034). In addition, there was a higher frequency of intestinal type of Lauren classification (66.7%) in high MEK group compared to low MEK (20.5%) and intermediate MEK group (36.6%) (*P* = 0.012). There was no significant difference of age, sex and tumor stage. Long-term follow up data (more than 5 years) was available for the patients of the discovery set (*n* = 38). Disease-related death rate was 37% in low, 61.1% in intermediate, and 66.7% in high group.

**Table 2:** Clinicopathologic characteristics of Korean GC patients according to the MEK signature

Variable	MEK signature			
	Low (*n* = 39)	Intermediate (*n* = 71)	High (*n* = 15)	*P* Value
KRAS mutation/ amplification				0.001
Wild	35 (89.7%)	57 (80.3%)	6 (40.0%)	
Mutant	4 (10.3%)	9 (12.7%)	4 (26.7%)	
Amp	0 (0%)	5 (7.0%)	5 (33.3%)	
Age				0.174
< 60	24 (61.5%)	41 (57.7%)	5 (33.3%)	
≥ 60	15 (38.5%)	30 (42.3%)	10 (66.7%)	
Sex				0.153
Male	23 (59.0%)	44 (62.0%)	13 (86.7%)	
Female	16 (41.0%)	27 (38.0%)	2 (13.3%)	
WHO type				0.034
W/D	1 (2.6%)	3 (4.2%)	1 (6.7%)	
M/D	4 (10.3%)	18 (25.4%)	6 (40.0%)	
P/D or Signet ring cell	33 (84.6%)	48 (67.6%)	7 (46.7%)	
Others	1 (2.6%)	2 (2.8%)	1 (6.7%)	
Lauren classification				
Intestinal	8 (20.5%)	26 (36.6%)	10 (66.7%)	0.012
Diffuse	31 (79.5%)	43 (60.6%)	5 (33.3%)	
Mixed	0 (0%)	2 (2.8%)	0 (0%)	
Stage				
I	0 (0%)	1 (1.4%)	1 (6.7%)	0.059
II	3 (7.7%)	9 (12.7%)	3 (20.0%)	
III	8 (20.5%)	20 (28.2%)	7 (46.7%)	
IV	28 (71.8%)	41 (57.7%)	4 (26.7%)	

## DISCUSSION

MEK inhibition in combination with chemotherapy has shown beneficial effects in *KRAS* mutant lung cancer and biliary tract cancer [8, 12]. This strategy could be potentially adapted to GC patients [13]. Therefore, development of a robust diagnostic tool indicative of RAS pathway activity is needed for selection of patients who can benefit from MEK inhibition.

RAF-MEK-ERK pathway has profound effects on proliferative, apoptotic, and differentiation pathways [14]. Mutations occurring in RAF-MEK-ERK pathway can lead to uncontrolled regulation and aberrant signaling [14]. However, the regulation of this pathway is a rather complex process and there are also many tumor suppressor proteins interacting with this pathway such as PTEN, RKIP, PP2A, DUSP5, DUSP6, TSC1, and TSC2 [14]. Expression of activated Ras in gastric chief cells of mice resulted in metaplastic lineage transitions in stomach, which implicated that RAS signaling pathway inhibition may reverse preneoplastic metaplasia in the stomach [15].

Selumetinib (AZD 6244, ARRY-142886) is a potent, orally active inhibitor of mitogen-activated protein/extracellular signal-regulated kinase (ERK) kinase [1]. Previous *in vivo* and *in vitro* studies demonstrated a tendency toward sensitivity to selumetinib in tumors harboring *KRAS* or *BRAF* mutations compared with those without those mutations [1, 2]. However, a recent study has shown that selumetinib response does not have an absolute correlation with mutational status of *BRAF*, *RAS*, or *PIK3CA* [2].

Although the development and clinical trial of selumetinib has focused on *KRAS*-mutant non-small-cell lung cancer or *BRAF*-mutant melanoma, it is not clear that MEK inhibitors are specific for *KRAS*-mutant cancers [5]. The response rates to MEK inhibitor in clinical trials has varied. A recent study has shown that patients harbouring *KRAS* G12C or G12V mutations showed better response for selumetinib plus docetaxel compared with other *KRAS* mutations [16]. On the other hand, of twenty patients who showed response in phase II study of selumetinib in metastatic biliary tract cancers, only two patients had *KRAS* mutations [12]. Another study also failed to show improvement in objective response rate or progression-free survival with combination therapy of selumetinib and erlotinib over monotherapy in *KRAS* mutant and *KRAS* wild-type advanced lung cancer [17].

Clinically, it is important to develop an accurate and robust method indicative of RAS pathway activity to stratify potential patients who can benefit from selumetinib treatment. Previous *in vivo* and *in vitro* studies have suggested that gene expression signature would provide a better measure of RAS activity in cancer cells than mutation analysis [9]. In a previous data set, tumors with *KRAS* mutations had high signature scores [9]. However, a significant number of *KRAS* wild-type cell lines and tumors exhibited high RAS pathway signature scores, suggesting that these samples have upregulated RAS signaling through another mechanism [9]. In our study, we performed nanostring assays to evaluate pathway-associated gene expression and calculate MEK signature using six genes, *DUSP4*, *DUSP6*, *ETV4*, *ETV5*, *PHLDA1*, and *SPRY2*, in advanced GC patients. Our results showed that *KRAS* altered tumors have a tendency to have high MEK signature. However, the relationship between MEK signature and *KRAS* status was not absolute in our study. There is a subset of *KRAS* wild type GCs which revealed high MEK signature which is in agreement with previous findings that the RAS pathway signature is a high sensitivity but low specificity predictor of *KRAS* mutation status [9]. *PIK3CA* is known to mediate resistance to MEK inhibitor [2, 18]. In our cohort, no tumor harboring *PIK3CA* mutation had high MEK signature. A phase II selumetinib/docetaxel trial is currently ongoing for second-line metastatic GC patients as part of an umbrella trial with patient selection based on high vs low MEK signature or RAS gene alterations (clinicaltrials.gov NCT#02448290).

GC is the second most common cause of cancer-related deaths worldwide, and the prognosis of advanced GC is still poor [19]. To date, only trastuzumab along with chemotherapy showed positive survival outcomes among many targeted therapy trials [13, 20]. One study has reported a potential relationship between *KRAS* amplification, the activation of *KRAS* signaling pathway, and cell growth in GC [21]. *KRAS* mutation was more frequently found in intestinal type, according to the Lauren classification [22, 23]. Our study showed that MEK high signature was more prevalent in well-differentiated and moderately-differentiated tumor type and intestinal type by Lauren classification. In addition to *KRAS* mutation, MEK1 S72G mutation [24], which activates ERK1/2 and enhances tumorigenicity, has been shown to be hypersensitive to MEK inhibitors in GC. The correlation between MEK signature and MEK1 mutation should be investigated in future studies.

In conclusion, 6.9% of metastatic GC score high for expression of the MEK signature assessed by nanostring assay and have tendency to be enriched for *KRAS* genomic alterations and intestinal type tumors by Lauren classification. The correlation between MEK signature, KRAS alteration and treatment response to selumetinib should be validated in prospective clinical trials.

## MATERIALS AND METHODS

### Selumetinib sensitivity and MEK signature scoring in GC cell lines

MTS was used to determine cell proliferation. Cells were seeded in 96-well plates at a density to allow for logarithmic growth during the 72-hour assay and then incubated overnight at 37°C, 5% CO_2._ Cells were treated with concentrations of selumetinib ranging from 30 to 0.003 μmol/L for 72 hours. For the MTS endpoint, cell proliferation was measured by the CellTiter AQueous Non-Radioactive Cell Proliferation Assay (Promega) reagent as per manufacturer's instructions. Absorbance was measured with a Tecan Ultra instrument. Expression of MEK and Cres signature genes was determined by RNA sequencing. Total RNA was extracted from cell lines and mRNA was enriched using the oligo dT magnetic beads. cDNA was synthesized from fragmented and size selected mRNA (~200 bp). Sequencing adaptors were ligated to the fragments and PCR amplified before sequenced using Illumina HiSeq 2000's pair-end sequencing technology (2 × 90 bp). Roughly ~120 million reads were obtained for each cell line.

Sensitivity to MEK inhibition with selumetinib was defined as a GI50 under 3 μM, and further divided into partial (TGI not reached within 30 mM concentration range tested) and extreme (TGI< 5 μM) sensitivity. Resistant cell lines showed GI50> 20 μM and did not reach TGI within 30 μM concentration range tested. MEK signature was scored from RNAseq as the mean of gene centric RPKM for the 18 MEK signature genes [2]. Association between MEK signature score and categorical sensitivity to selumetinib was determined using ANOVA.

### Patient population

This study was conducted in two cohorts of Asian GC patients, which consisted of a Korean cohort (*n* = 125) and a Vietnamese cohort (*n* = 93). For the training set in the Korean cohort, positive control (*KRAS* altered) and negative control (*KRAS* wild) cases were selected from 312 surgically resected GC samples that had been sequenced as previously reported [11]. Positive control group (*n* = 17) consisted of 8 *KRAS* amplified tumors and 9 *KRAS* mutated tumors by target sequencing. Negative control group (*n* = 21) was confirmed to have no *KRAS* mutation and amplification. The remaining 87 of 125 specimens in the Korean cohort were from the GC molecular screening program, the VIKTORY molecular screening study (NCT# 02299648). Briefly, 87 patients had MEK signature scoring results from nanostring assay, in addition to the cancer panel sequencing results (either from Ion torrent or Illumina HiSeq). Clinicopathological information of Korean GC cohort, including age, sex, WHO histologic type, Lauren classification, clinical stage, and GC-associated survival data was collected. The study was approved by the institutional review board of Samsung Medical Center (IRB no. 2014-1-136). Written informed consent form was obtained from all patients before the study. All experimental procedures were carried out in accordance with guidelines approved by Samsung Medical Center.

### RNA preparation

Hematoxylin and Eosin stain was performed on one tumor section per patient and tumors were reviewed by a pathologist for tumor purity. Samples with < 50% tumor was discarded from the study. The tumor component was macro-dissected from 2 × 5 μm FFPET sections either from FFPET or fresh frozen samples and RNA was extracted using RNeasy FFPE Extraction kit (Qiagen) or QIAamp DNA Mini Kit (Qiagen, Hilden, Germany) according to the manufacturer's instructions. Sample RNA was quantified using Qubit 2.0 Flourometer with the Broad Range RNA kit using the standard protocol. Samples that had < 20 ng/μl total RNA were not tested in the nanostring assay. Where available, more tissue for these samples were ordered, re-extracted, and those 20 ng/ul or greater were tested in the nanostring assay.

### Nanostring assay and MEK signature calculation

Nanostring assays were performed by following the standard protocol ‘Setting up 12 nCounter Assays (MAN-C0003-03, 2008–2013). Hybridization incubations were performed between 17–18hrs. Cartridges were either read immediately or stored dark (in aluminum foil) at 4°C until ready to be read. All cartridges were read within 2 days of preparation, on the AZ GEN2 Digital Analyzer station with high resolution selected.

To enable assessment of sample MEK signature scores against previously defined cut-points, three reference standards, representing high, medium and low MEK signature scores were run in every assay. These reference samples were selected from a previous assessment of MEK signature scores using Nanostring in 197 commercially sourced Vietnamese Gastric cancer samples (unpublished data). Up to eight test samples and a background control sample (RNase free water instead of RNA in the hybridisation reaction) using the same reagents were ran alongside the reference samples. MEK signature score adjustment was undertaken by applying a linear transformation to the calculated MEK signatures for each sample. The transformation coefficients were calculated as the linear regression coefficients calculated from the fit of the reference sample MEK signature score values generated by Samsung Medical Center against those previously generated by AstraZeneca.

Each sample was normalized individually with the three reference samples using nSolver Analysis Software version 2.5. The normalized data was exported as Log2 data and then adjusted using the method described previously. Previously we published an 18-gene mRNA expression signature reproducibly predictive of MEK pathway output and response to MEK inhibition with selumetinib and subsequently demonstrated that a. 6-gene sub-signature shows enhanced reproducibility in cell lines, primary tumor explant *in vivo* models and fresh/fixed tissue samples, crossing tumor type and gene expression platforms (Dry *et al*. 2013). For each patient sample, the 6-gene MEK signature score was calculated by taking the arithmetic average of the normalized gene expression value (nSolver values) of the 6 MEK genes; DUSP4, DUSP6, ETV4, ETV5, PHLDA1, and SPRY2 . For each patient sample, the 6-gene MEK signature score was calculated by taking the arithmetic average of the normalized gene expression value (nSolver values) of the 6 MEK genes; DUSP4, DUSP6, ETV4, ETV5, PHLDA1, and SPRY2. Then, the adjusted MEK signature score was compared against the following two cut-off values; ‘MEK-low’ (25th percentile), 7.17 and ‘MEK-high’ (90th percentile), 8.08. Where adjusted MEK score for a patient was less than or equal to the MEK-low cut-off, the patient was considered as part of the MEK-low. Where adjusted MEK score for a patient was greater than or equal to the MEK-high cut-off, the patient was considered as part of the MEK-high.

### Statistical analysis

Statistical analysis was performed using SPSS version 18.0 (SPSS, Chicago, IL, USA). Contingency tables and Fisher's exact tests were used to correlate *KRAS* status and MEK signature results. One way Anova test was done to compare the distribution of MEK score according to *KRAS* and *PIK3CA* status. Contingency tables and Fisher's exact tests were also used to correlate MEK signature with *KRAS* status, age, sex, WHO tumor type, Lauren classification, and tumor stage. A *P*-value <0.05 was considered significant.
